# Supplementary material for: Stability of Mycotoxins in Individual Stock and Multi-Analyte Standard Solutions
Source: Toxins (Basel). 2020 Jan 30;12(2):94. doi: 10.3390/toxins12020094 (PMC7076964; doi:10.3390/toxins12020094)
Supplement: Supplementary file 1 [file toxins-12-00094-s001.pdf]

Supplementary Materials: Stability of Mycotoxins in Individual Stock and Multi-Analyte Standard Solutions

Mariya Kiseleva, Zakhar Chalyy, Irina Sedova and Ilya Aksenov

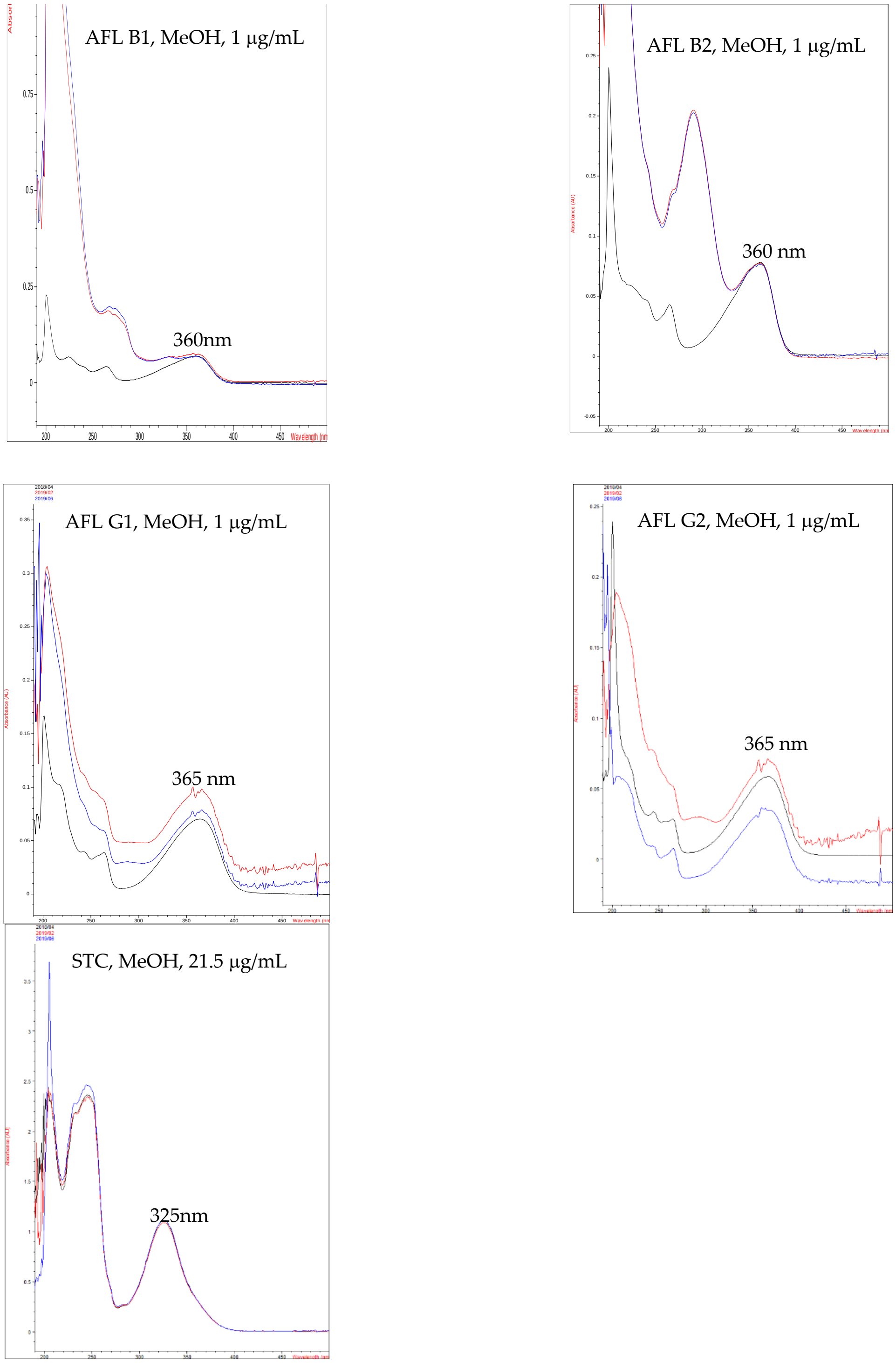

Figure 1. UV spectra of aflatoxins and sterigmatocystin individual standard solutions, recorded immediately after preparation (black line), after 10 (red line) and 14 (blue line) months of storage at -18°C.

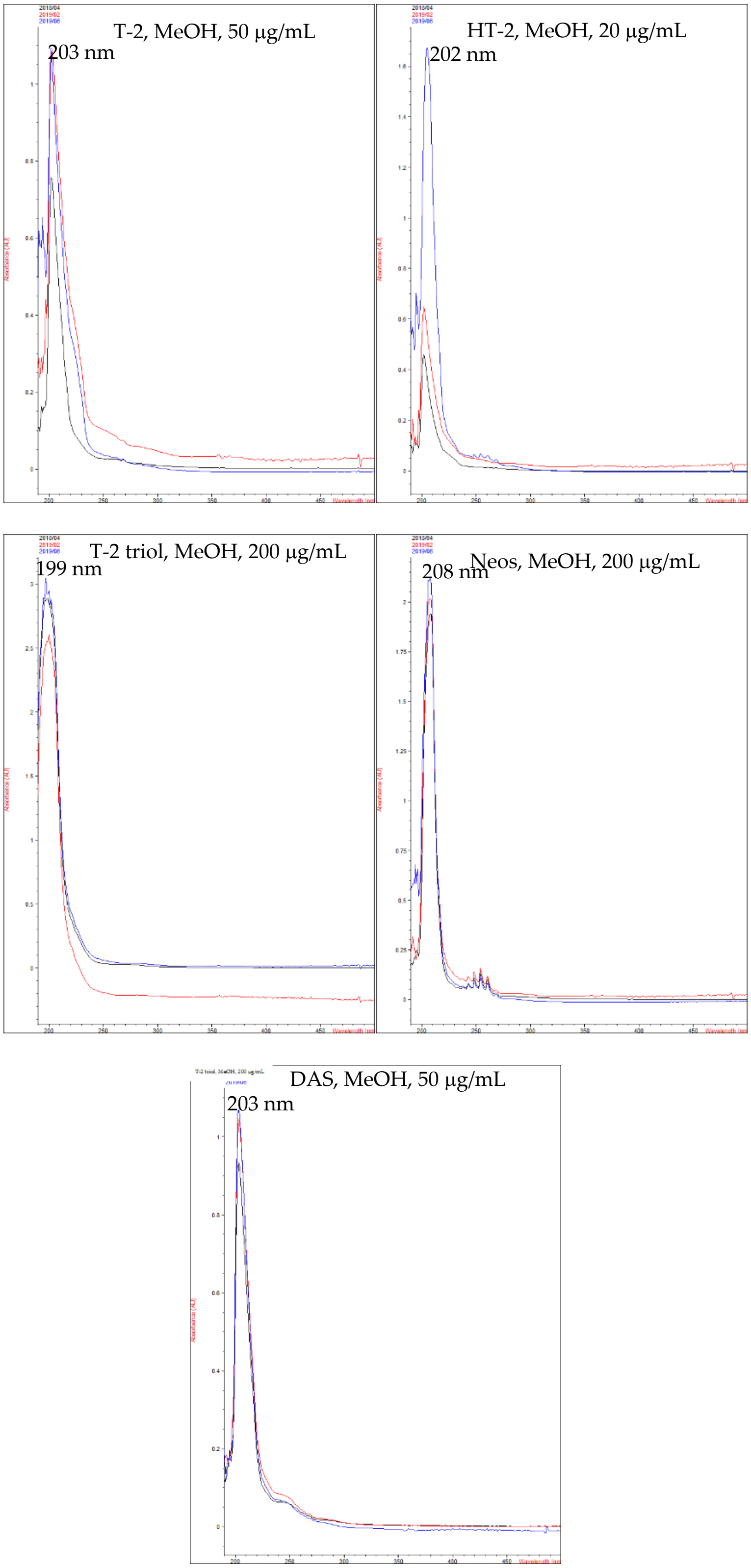

Figure 2. UV spectra of A-type trichothecenes individual standard solutions, recorded immediately after preparation (black line), after 10 (red line) and 14 (blue line) months of storage at -18°C.

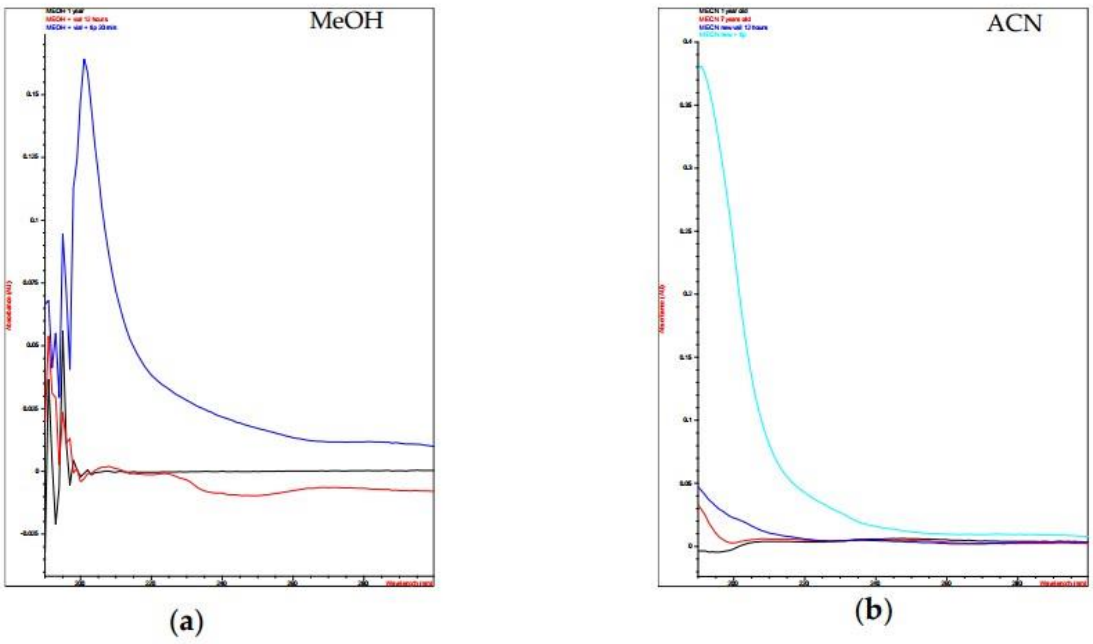

Figure 3. UV spectra of methanol (a): 1 year old solvent from the bottle (black line), the same solvent that has been stored in the vial for standard preparation for 12 hours (red line), methanol, in which pipette tip has been soaked for 20 minutes (blue line); UV spectra of acetonitrile (b): 1 year old solvent from the bottle (blue line); 7 years old solvent from the bottle (red line), 1 year old solvent that has been stored in the vial for standard preparation for 12 hours (blue line); acetonitrile, in which pipette tip has been soaked for 20 minutes (light-blue line).

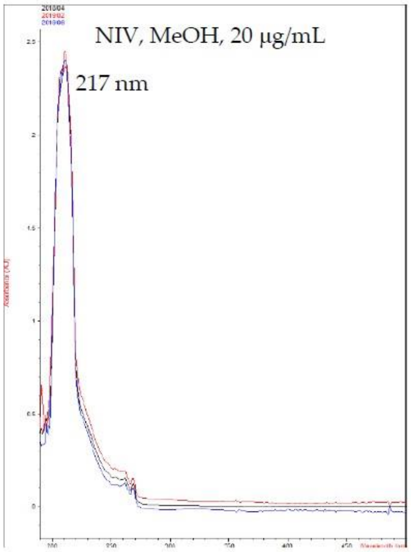
NIV, MeOH, 20 µg/mL
217 nm

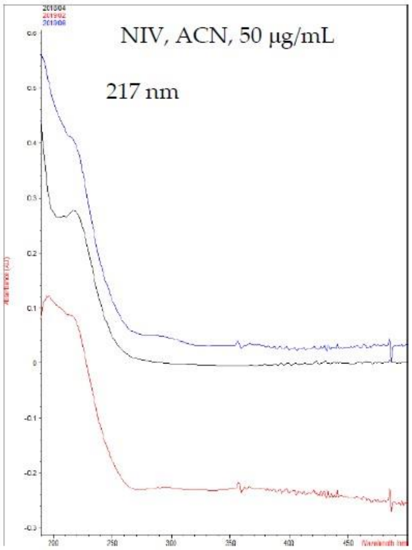
NIV, ACN, 50 µg/mL
217 nm

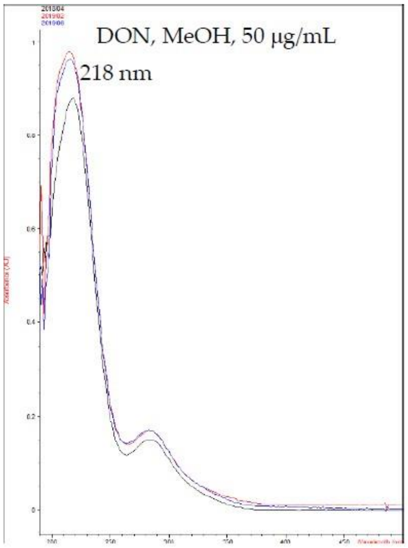
DON, MeOH, 50 µg/mL
218 nm

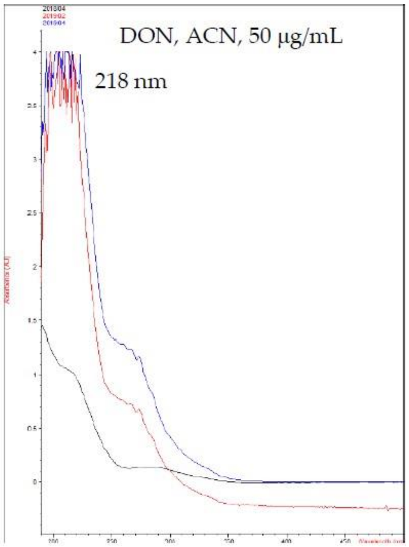
DON, ACN, 50 µg/mL
218 nm

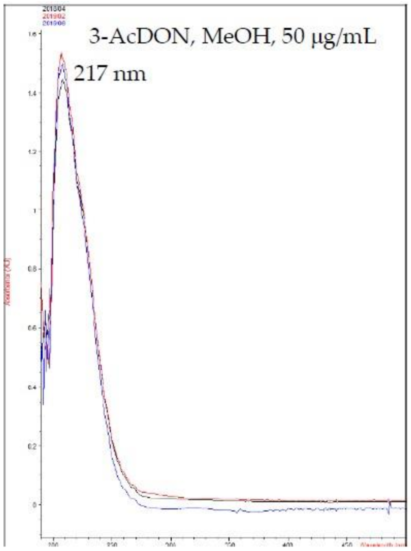
3-AcDON, MeOH, 50 µg/mL
217 nm

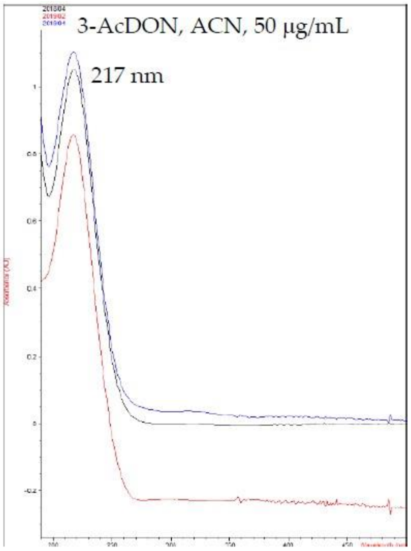
3-AcDON, ACN, 50 µg/mL
217 nm

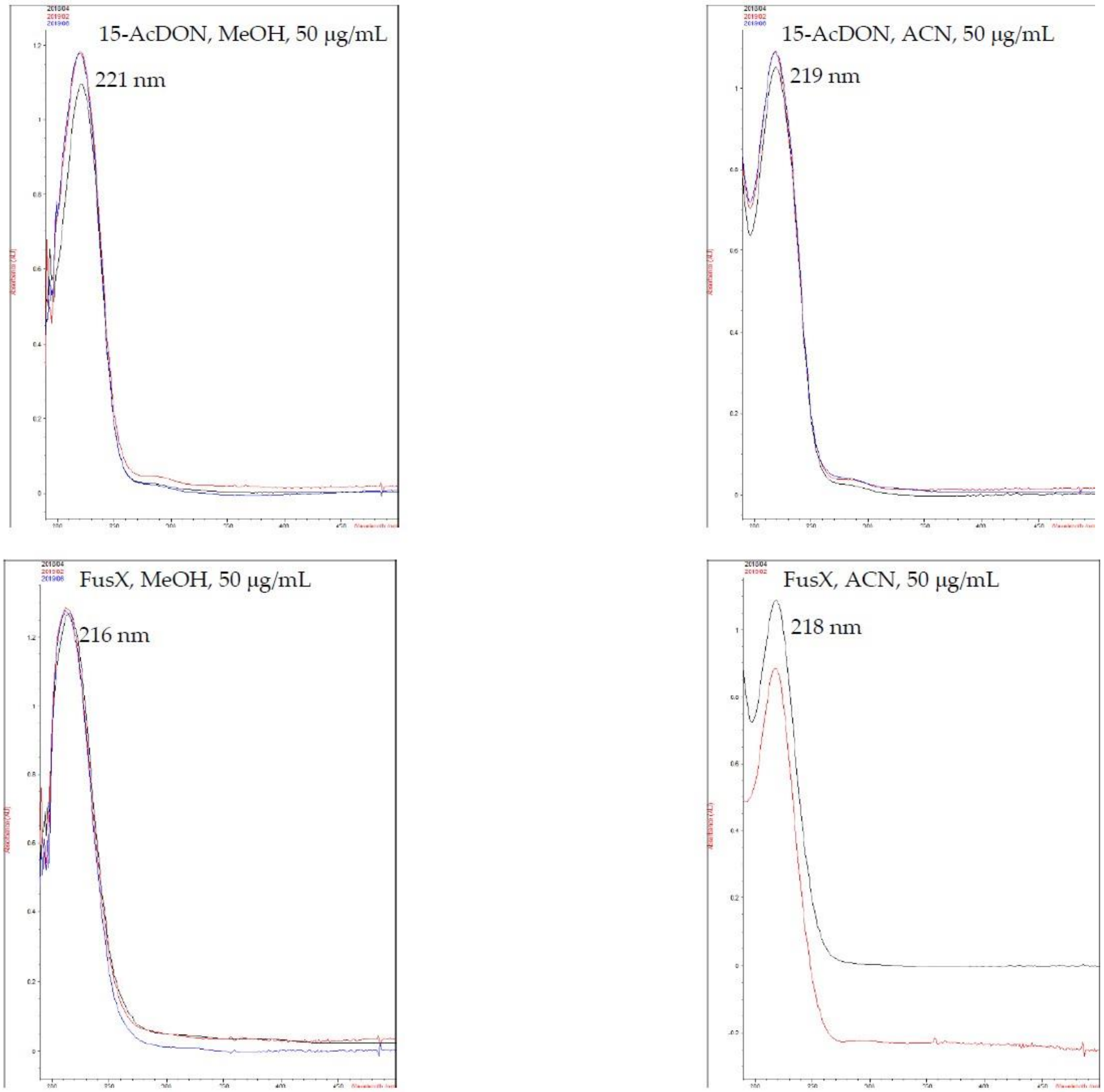

Figure 4. UV spectra of B-type trichothecenes individual standard solutions, recorded immediately after preparation (black line), after 10 (red line) and 14 (blue line) months of storage at -18°C.

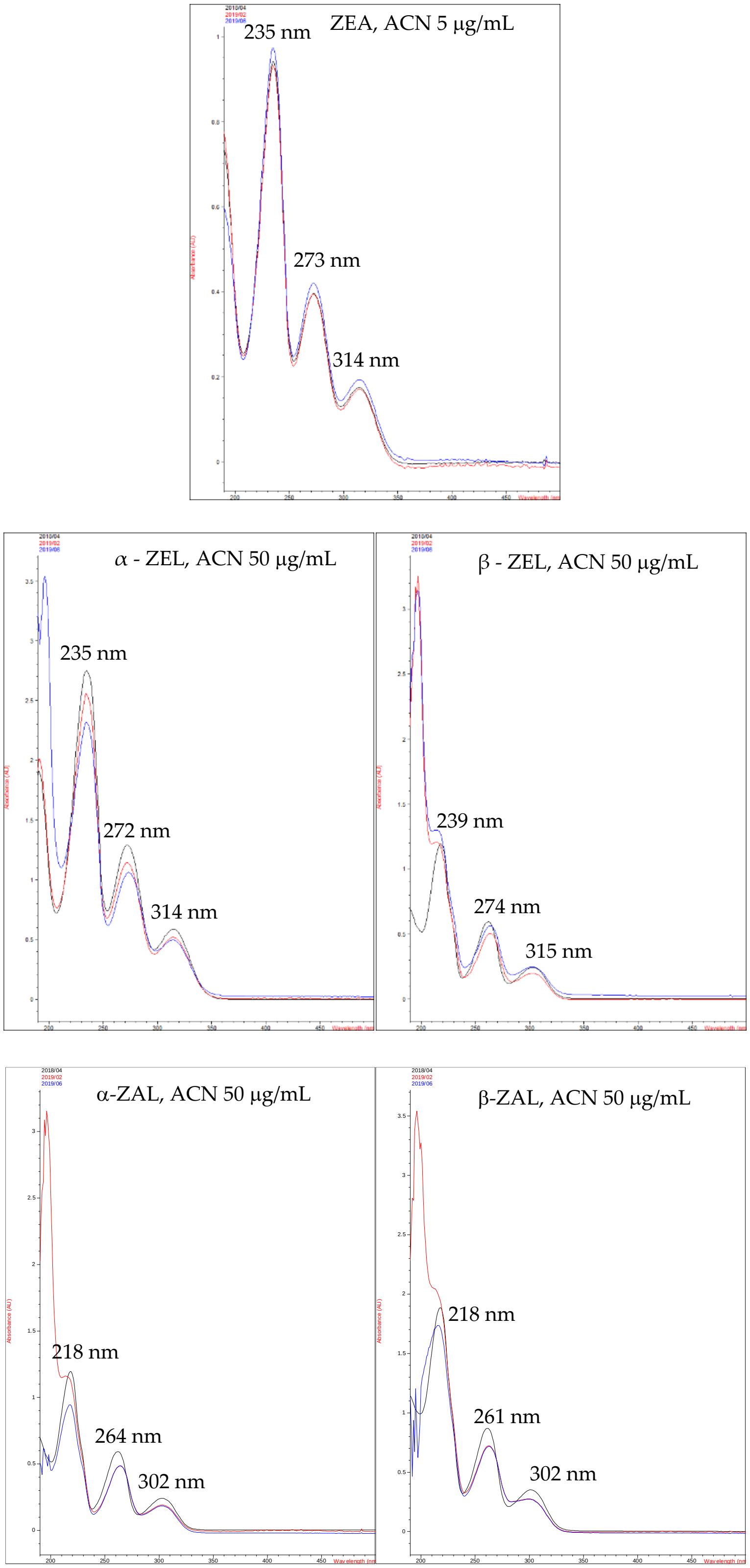

Figure 5. Spectra of ZEA and its derivatives in acetonitrile, recorded immediately after preparation (black line), after 10 (red line) and 14 (blue line) months of storage at -18°C.

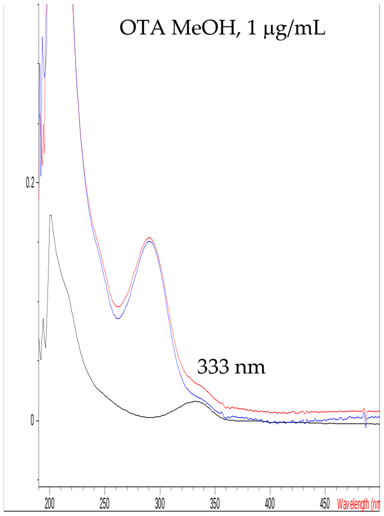

Figure 6. Spectra of OTA methanol individual standard solution, recorded immediately after preparation (black line), after 10 (red line) and 14 (blue line) months of storage at -18°C.

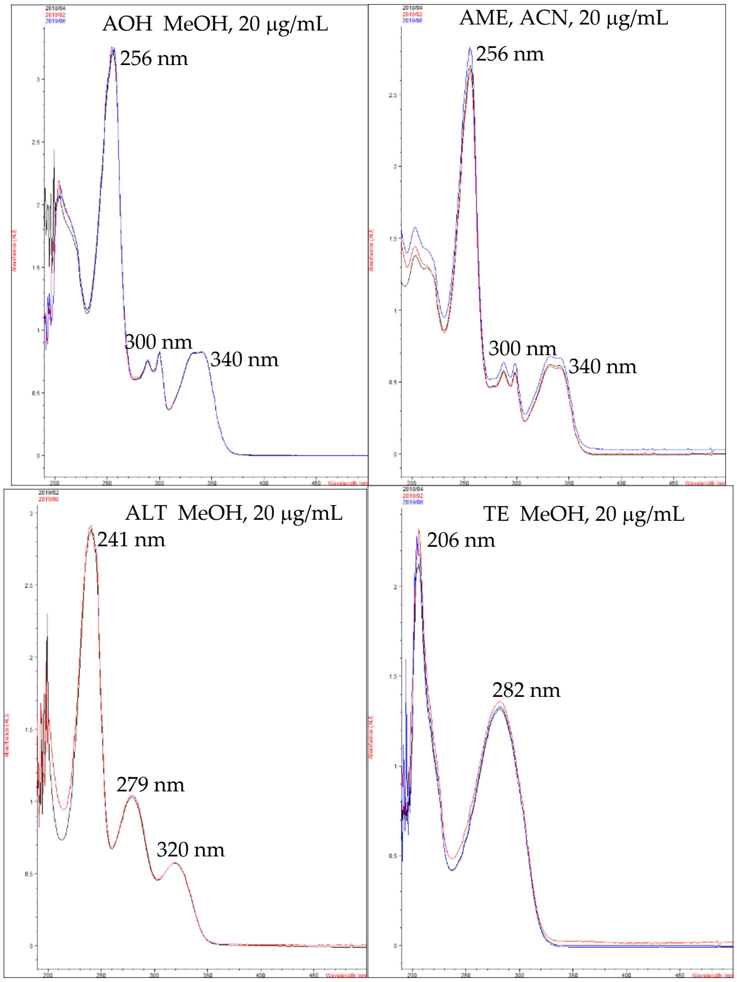

Figure 7. Spectra of *Alternaria* mycotoxins individual standard solutions, recorded immediately after preparation (black line), after 10 (red line) and 14 (blue line) months (AOH, AME, TE); after preparation (black line) and four (red line) months (ALT) of storage at -18°C.

nniatins A and B, beauvericin

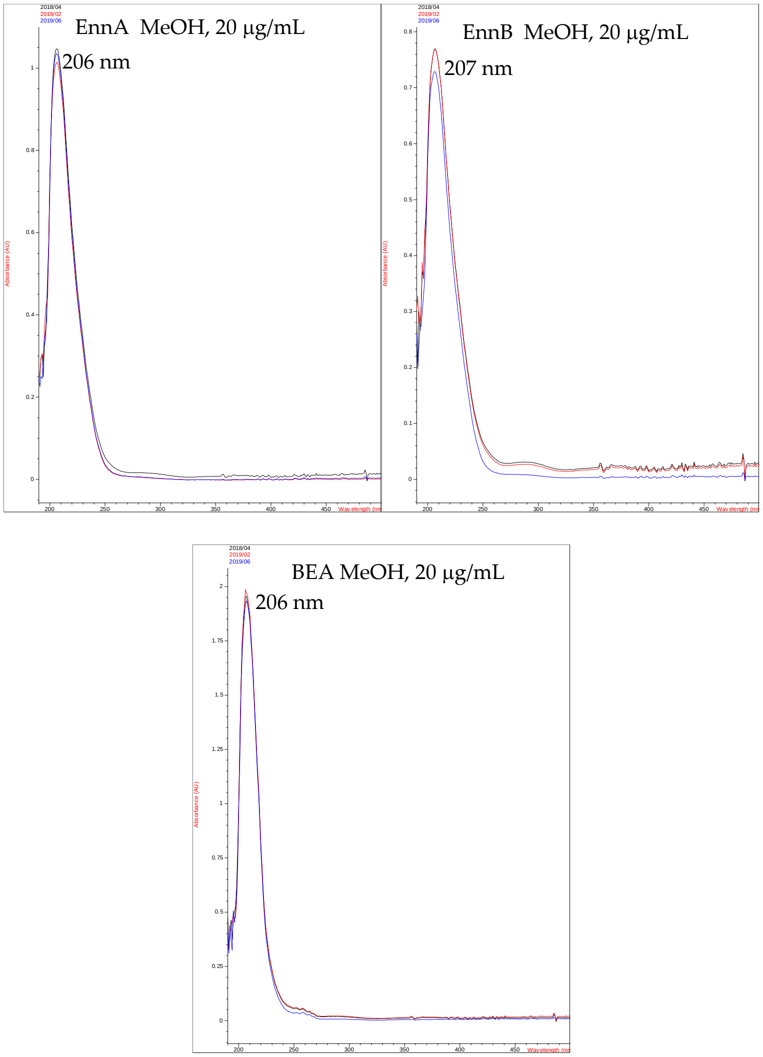

Figure 8. Spectra of EnnA, EnnB and BEA methanol individual standard solution, recorded immediately after preparation (black line), after 10 (red line) and 14 (blue line) months of storage at -18°C.

Moniliformin, mycophenolic acid, citrinin, citreoviridin, cyclopyazonic acid

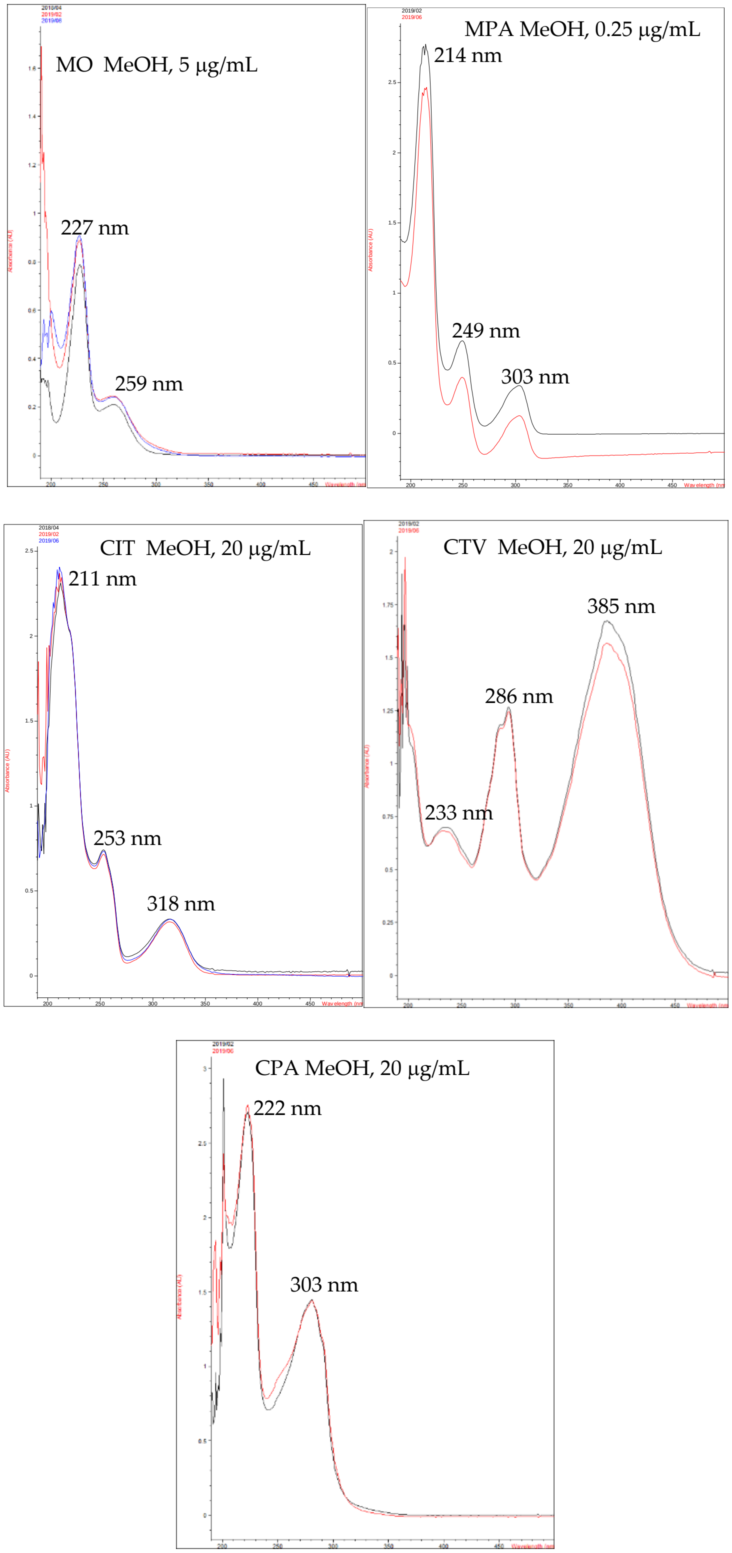

Figure 9. Spectra of mycotoxins individual standard solutions, recorded immediately after preparation (black line), after 10 (red line) and 14 (blue line) months (MO, CIT); after preparation (black line) and four (red line) months (MPA, CTA, CTV) of storage at -18°C.

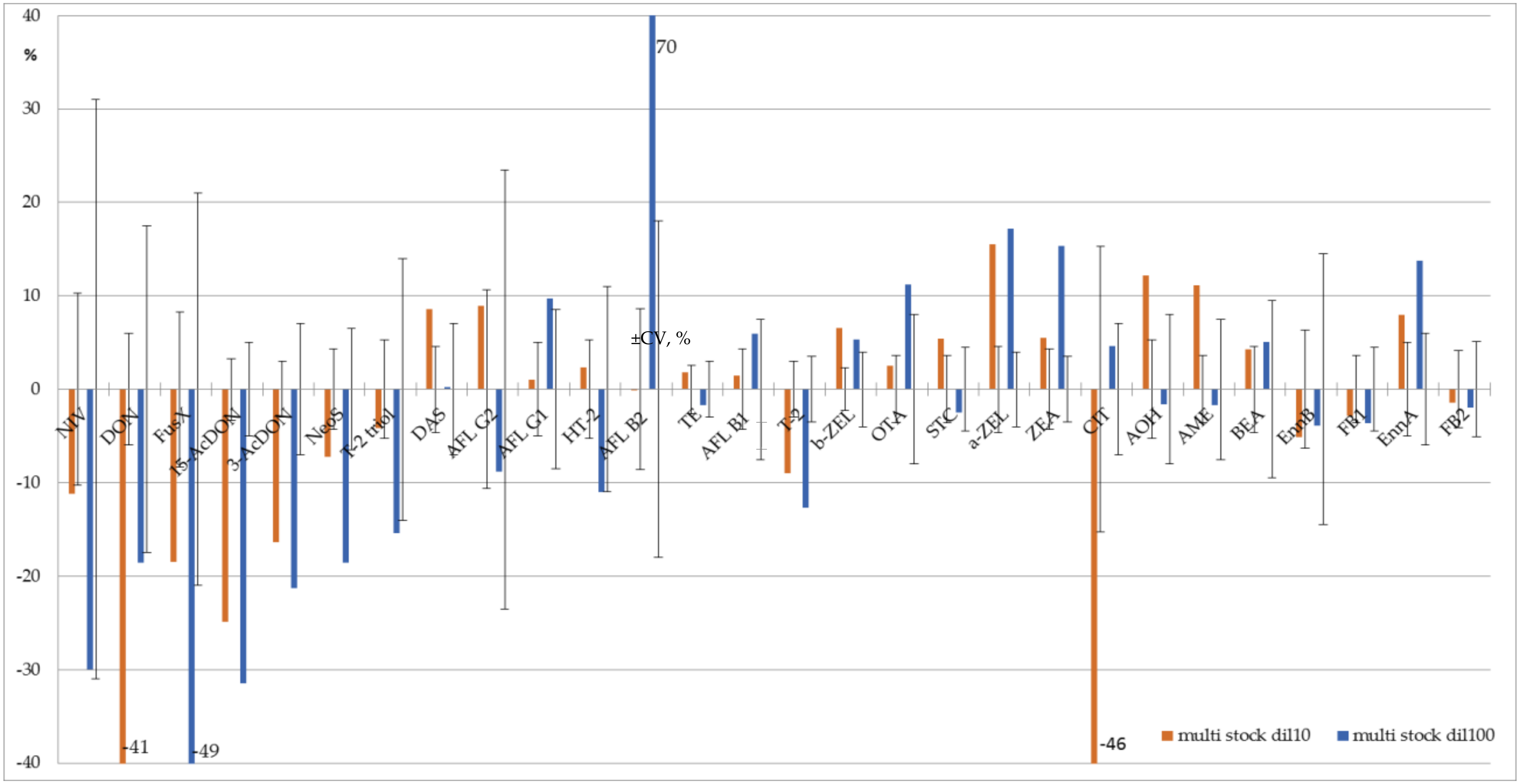

Figure 10. Suppression/enhancement of MS/MS analytical signal within 75 hours of stability monitoring of multi-mycotoxin stock standard solution diluted 10- and 100-fold (brown and blue correspondingly). Error bars indicate average coefficient of variation of analytical signal of mycotoxin in 10- and 100-fold diluted multi-analyte standard solution. Mycotoxins are presented in their elution order.

Table 1. SRM table for studied mycotoxins.

Compound	Retention Time, min	ΔRT, min	Polarity	Precursor (m/z)	Product (m/z)	Collision Energy, V	RF Lens, V
NIV_1	1.2	1	Negative	357.382	281.111	10.25	86
DON_1	1.7	1	Positive	297.083	159.111	31.44	100
DON_2_Q	1.7	1	Positive	297.083	249.111	10.61	100
DON_3	1.7	1	Positive	297.083	267.111	17.89	100
FusX_1	2.2	2	Positive	355.402	175.04	20.42	103
FusX_2	2.2	2	Positive	355.402	229.111	16.02	103
FusX_3_Q	2.2	2	Positive	355.402	247.04	12.33	103
15AcDON_1_Q	3.5	3	Positive	339.144	137.11	10	97
15AcDON_2	3.5	3	Positive	339.144	231.125	12.88	97
15AcDON_3	3.5	3	Positive	339.144	261.097	10.25	97
3AcDON_1_Q	3.8	3	Positive	339.144	203.04	17.08	97
3AcDON_2	3.8	3	Positive	339.144	213.111	17.63	97
3AcDON_3	3.8	3	Positive	339.144	231.125	12.68	97
NeoS_1	5.5	2	Positive	400.197	197.169	16.72	79
NeoS_2	5.5	2	Positive	400.197	203.097	17.18	79
NeoS_3_Q	5.5	2	Positive	400.197	215.097	16.62	79
T-2 Triol_1	5.5	2	Positive	400.233	145.183	25	76
T-2 Triol_2_Q	5.5	2	Positive	400.233	263.097	13	76
T-2 Triol_3	5.5	2	Positive	400.233	365.222	10	76
DAS_1	6.0	2	Positive	384.202	229.11	15	89
DAS_2	6.0	2	Positive	384.202	247.11	14	89
DAS_3_Q	6.0	2	Positive	384.202	307.151	10.25	89
AflaG2_1	6.5	2	Positive	331.081	189.111	41.4	170
AflaG2_2_Q	6.5	2	Positive	331.081	245.111	29.67	170
AflaG2_3	6.5	2	Positive	331.081	285.111	27.29	170
AflaG1_1	7.1	2	Positive	329.066	199.986	40.59	150
AflaG1_2_Q	7.1	2	Positive	329.066	243.04	26.08	150
AflaG1_3	7.1	2	Positive	329.066	311.04	21.02	150
HT-2_Na_1	7.4	2	Positive	447.375	284.923	20	163
HT-2_Na_2_Q	7.4	2	Positive	447.375	345.375	17	163
AflaB2_1	7.5	2	Positive	315.086	243.04	39.38	170
AflaB2_2_Q	7.5	2	Positive	315.086	259.04	29.01	170
AflaB2_3	7.5	2	Positive	315.086	287.111	25.62	170
TE_1	7.7	2	Positive	415.284	256.222	29.37	129
TE_2	7.7	2	Positive	415.284	302.169	13.13	129
TE_3_Q	7.7	2	Positive	415.284	312.151	19.3	129
AflaB1_1	8.0	2	Positive	313.071	213.04	44.58	166
AflaB1_2	8.0	2	Positive	313.071	241.04	37.25	166
AflaB1_3_Q	8.0	2	Positive	313.071	285.111	22.44	166
T-2_Na_1	9.3	2	Positive	489.138	244.954	23	138

T-2_Na_2_Q	9.3	2	Positive	489.138	326.887	23	138
T-2_Na_3	9.3	2	Positive	489.138	387.375	20.5	138
CTV_3	9.5	2	Positive	403.2	285	10	45
CTV_2_Q	9.5	2	Positive	403.2	297	10	45
CTV_1	9.5	2	Positive	403.2	315	10	45
b-ZEL_1	10.3	4	Positive	321.32	189.1	20	88
b-ZEL_2_Q	10.3	4	Positive	321.32	285.1	10	88
b-ZEL_3	10.3	4	Positive	321.32	303.1	10	88
OTA_1	11.0	2	Positive	404.09	221	34.72	123
OTA_2_Q	11.0	2	Positive	404.09	239	23.5	123
OTA_3	11.0	2	Positive	404.09	358.111	14.04	123
STC_1	11.8	2	Positive	325.071	253.04	43.67	152
STC_2_Q	11.8	2	Positive	325.071	281.04	36.24	152
STC_3	11.8	2	Positive	325.071	310.058	24.01	152
a-ZEL_1	12.1	4	Positive	321.12	177.11	19	65
a-ZEL_2_Q	12.1	4	Positive	321.12	285.11	10	65
a-ZEL_3	12.1	4	Positive	321.12	303.22	11	65
ZEA_1	12.1	2	Positive	319.2	185	15	90
ZEA_2_Q	12.1	2	Positive	319.2	283.1	10	90
ZEA_3	12.1	2	Positive	319.2	301.2	10	90
CIT_1	12.2	4	Positive	251.154	115.151	52.32	93
CIT_2_Q	12.2	4	Positive	251.154	191.111	25.47	93
CIT_3	12.2	4	Positive	251.154	205.111	26.53	93
AOH_1	12.8	2	Positive	259.322	128.125	44	100
AOH_2_Q	12.8	2	Positive	259.322	185.097	31	100
AOH_3	12.8	2	Positive	259.322	213.054	27	100
AME_1	14.2	2	Positive	273.1	128.1	60	150
AME_2	14.2	2	Positive	273.1	185.1	40	150
AME_3_Q	14.2	2	Positive	273.1	230	30	150
BEA_1	15.0	4	Positive	801.44	134.222	54	215
BEA_2	15.0	4	Positive	801.44	244.22	32	215
BEA_3	15.0	4	Positive	801.44	262.22	30	215
EnnB_1_Q	15.1	2	Positive	657.58	214.22	31	142
EnnB_2	15.1	2	Positive	657.58	527.4	27	142
EnnB_3	15.1	2	Positive	657.58	640.556	17	142
FB1_1_Q	15.3	2	Positive	722.484	334.333	39.98	217
FB1_2	15.3	2	Positive	722.484	352.405	35.79	217
FB1_3	15.3	2	Positive	722.484	704.502	27.85	217
EnnA_1_Q	16.0	2	Positive	682.664	210.222	24	255
EnnA_2	16.0	2	Positive	682.664	228.22	24	255
EnnA_3	16.0	2	Positive	682.664	229.11	21	255
FB2_1	19.0	2	Positive	706.488	318.405	36.49	150

FB2_2_Q	19.0	2	Positive	706.488	336.405	35.74	150
FB2_3	19.0	2	Positive	706.488	354.405	33.66	150

Table 2. Suppression/enhancement of MS/MS analytical signal (SSE) within 75 hours of stability monitoring of multi-mycotoxin stock standard solution diluted 10- and 100-fold (SSE (dil10) and SSE(dil100) correspondingly) and average coefficients of variation of analytical signal of mycotoxin in 10- and 100-fold diluted multi-analyte standard solution (CVaver (dil10) and CVaver(dil100) correspondingly).

MT	SSE(dil10)	CVaver(dil10)	SSE(dil100)	CVaver(dil100)
NIV	-11,2	10,3	-30	31
DON	-41,0	6	-18,6	17,5
FusX	-18,5	8,3	-48,5	21
15-AcDON	-24,9	3,3	-31,5	5
3-AcDON	-16,4	3	-21,3	7
NeoS	-7,2	4,3	-18,6	6,5
T-2 triol	-4,2	5,3	-15,4	14
DAS	8,6	4,6	0,2	7
AFL G2	8,9	10,6	-8,8	23,5
AFL G1	1	5	9,7	8,5
HT-2	2,3	5,3	-11	11
AFL B2	-0,1	8,6	70	18
TE	1,8	2,6	-1,7	3
AFL B1	1,5	4,3	5,9	7,5
T-2	-9	3	-12,7	3,5
b-ZEL	6,6	2,3	5,3	4
OTA	2,5	3,6	11,2	8
STC	5,4	3,6	-2,5	4,5
a-ZEL	15,5	4,6	17,2	4
ZEA	5,5	4,3	15,3	3,5
CIT	-46,3	15,3	4,6	7
AOH	12,2	5,3	-1,6	8
AME	11,1	3,6	-1,7	7,5
BEA	4,3	4,6	5,1	9,5
EnnB	-5,1	6,3	-3,9	14,5
FB1	-2,8	3,6	-3,6	4,5
EnnA	8	5	13,8	6
FB2	-1,4	4,1	-2	5,1
